# Correlations between cytoplasmic CSE1L in neoplastic colorectal glands and depth of tumor penetration and cancer stage

**DOI:** 10.1186/1479-5876-11-29

**Published:** 2013-01-31

**Authors:** Cheng-Jeng Tai, Tzu-Cheng Su, Ming-Chung Jiang, Hung-Chang Chen, Shing-Chuan Shen, Woan-Ruoh Lee, Ching-Fong Liao, Ying-Chun Chen, Shu-Hui Lin, Li-Tzu Li, Ko-Hung Shen, Chung-Min Yeh, Kun-Tu Yeh, Ching-Hsiao Lee, Hsin-Yi Shih, Chun-Chao Chang

**Affiliations:** 1 Department of Internal Medicine, School of Medicine, College of Medicine, Taipei Medical University, Hospital, No.250, Wu-Hsing St., Taipei 11031, Taiwan; 2 Division of Hematology and Oncology, Department of Internal Medicine, Taipei Medical University Hospital, No.250, Wu-Hsing St., Taipei 11031, Taiwan; 3 Department of Pathology, Changhua Christian Hospital, 135 Nan-Hsiao St., Changhua, 50006 Taiwan; 4 Division of Colorectal Surgery, Changhua Christian Hospital, No. 135 Nan-Hsiao St., Changhua 50006, Taiwan; 5 Jen-Teh Junior College of Medicine, Nursing and Management, No.79-9, Shalunhu, Houlong Township, Miaoli 35664, Taiwan; 6 Graduate Institute of Medical Sciences, College of Medicine, Taipei Medical University, No.252, Wu-Hsing St., Taipei 11031, Taiwan; 7 Institute of Cellular and Organismic Biology, Academia Sinica, No. 128 Academia Road, Section 2, Nankang, Taipei 11529, Taiwan; 8 School of Medicine, Chung Shan Medical University, No.110, Sec.1, Jianguo N.Rd., Taichung 40201, Taiwan; 9 Institute of Clinical Medicine, Kaohsiung Medical University, No.100, Shih-Chuan 1st Road, Kaohsiung 80708, Taiwan; 10 Division of Gastroenterology and Hepatology, Department of Internal Medicine, Taipei Medical University Hospital, No.250, Wu-Hsing St., Taipei 11031, Taiwan

**Keywords:** Colorectal cancer, CSE1L, Cytoplasm, Invasion, Metastasis, Nucleus

## Abstract

**Background:**

Colorectal carcinomas spread easily to nearby tissues around the colon or rectum, and display strong potential for invasion and metastasis. CSE1L, the chromosome segregation 1-like protein, is implicated in cancer progression and is located in both the cytoplasm and nuclei of tumor cells. We investigated the prognostic significance of cytoplasmic vs. nuclear CSE1L expression in colorectal cancer.

**Methods:**

The invasion- and metastasis-stimulating activities of CSE1L were studied by *in vitro* invasion and animal experiments. CSE1L expression in colorectal cancer was assayed by immunohistochemistry, with tissue microarray consisting of 128 surgically resected specimens; and scored using a semiquantitative method. The correlations between CSE1L expression and clinicopathological parameters were analyzed.

**Results:**

CSE1L overexpression was associated with increased invasiveness and metastasis of cancer cells. Non-neoplastic colorectal glands showed minimal CSE1L staining, whereas most colorectal carcinomas (99.2%, 127/128) were significantly positive for CSE1L staining. Cytoplasmic CSE1L was associated with cancer stage (*P*=0.003) and depth of tumor penetration (*P*=0.007). Cytoplasmic CSE1L expression also correlated with lymph node metastasis of the disease in Cox regression analysis

**Conclusions:**

CSE1L regulates the invasiveness and metastasis of cancer cells, and immunohistochemical analysis of cytoplasmic CSE1L in colorectal tumors may provide a useful aid to prognosis.

## Background

Colorectal cancer (CRC) shows a high rate of metastasis and is one of the most lethal cancers worldwide. Metastasis, especially occult metastasis, contributes considerably to the challenge of defining the prognosis for a patient with this disease. Approximately 60% of patients who undergo curative resection experience local recurrence or distant metastases [1]. The primary treatment of CRC is surgical resection of the primary tumor and possibly the regional lymph nodes; this may be combined with chemotherapy, depending on the depth of tumor penetration and the disease stage [2]. The development and progression of a tumor is controlled by oncogenes and tumor suppressor genes. Alterations in the expression of any of these genes in a tumor may correlate with the clinical-pathological characteristics and behavior of CRC. Thus, examination of these gene expressions may be useful for prognosis of the disease.

Chromosome segregation 1-like protein (CSE1L) is the human homologue of CSE1, the yeast chromosome segregation protein [3]. CSE1L is highly expressed in most cancers, and its expression has been shown to correlate with cancer progression [4-12]. CSE1L is located in both the cytoplasm and the nuclei of cells. Nuclear CSE1L regulates the transcriptional activity of the p53 protein, a major tumor suppressor protein [13,14]. Cytoplasmic CSE1L is associated with microtubules; this association has been shown to stimulate the extension of invadopodia (invasive feet) and to enhance the migration of tumor cells [15]. Therefore, both nuclear and cytoplasmic CSE1L are implicated in cancer progression. We analyzed the relationships between CSE1L cytoplasmic/nuclear distribution and the clinical-pathological characteristics of CRC. The findings showed that high CSE1L cytoplasmic expression was associated with the depth of tumor penetration and disease stage. We concluded that cytoplasmic CSE1L plays a role in the invasion and metastasis of CRC; furthermore, immunohistochemical analysis of CSE1L distribution in a tumor provides a useful ancillary tool for the prognosis of CRC.

## Methods

### Cells, Vectors, and DNA Transfection

B16F10 melanoma cells and COLO 205 colon cancer cells were obtained from the American Type Culture Collection (Manassas, VA, USA). The human HT-29 colon cancer cells transfected with vectors expressing CSE1L (i.e. the HT-29-CSE1L cells) and HT-29 cells transfected with the empty vectors (i.e. the HT-29-EV cells) were established previously [16]. Cells were cultured in Dulbecco’s modified Eagle’s medium (DMEM) supplemented with 10% heat-inactivated fetal bovine serum (FBS), 100 units/mL of penicillin, 100 mg/mL of streptomycin, and 2 mmol/L of glutamate at 37°C under a humidified 5% CO_2_ atmosphere. B16F10 cells and COLO 205 cells were separately transfected with pcDNA-CSE1L [16], a eukaryotic expression vector capable of expressing CSE1L, using the Lipofectamine plus reagent (Invitrogen, Carlsbad, CA, USA). Transfected cells were selected with G418 for 3 wk. Multiple drug-resistant colonies (>100) were pooled and amplified in mass culture. The transfected cells were maintained in media containing 200 mg/mL of G418; for the experiments, cells were cultured in medium without G418.

### Western blotting

Cells were harvested using a rubber policeman and were washed with ice-cold phosphate buffered saline (PBS). They were then lysed in an ice-cold radioimmunoprecipitation assay (RIPA) buffer (25 mM Tris-HC1, pH 7.2, 0.1% sodium dodecyl sulfate [SDS], 0.1% Triton X-100, 1% sodium deoxycholate, 150 mM NaC1, 1 mM ethylenediaminetetraacetic acid [EDTA], 1 mM sodium orthovanadate, 1 mM phenylmethanesulfonyl fluoride, 10 μg/mL aprotinin, and 5 μg/ml leupeptin). Protein concentration was determined using a BCA protein assay kit (Pierce, Rockford, IL, USA). Samples were resolved with SDS-polyacrylamide gel electrophoresis. Proteins were transferred to nitrocellulose membranes (Amersham Pharmacia, Buckinghamshire, UK), and immunoblotting was performed by enhanced chemiluminescence (ECL) using an ECL Plus Western Blotting Detection System (GE Healthcare, Little Chalfont, Buckinghamshire, UK) with the anti-CSE1L (clone 24) antibody (Santa Cruz Biotechnology, Santa Cruz, CA, USA). Levels of β-actin were assayed with anti-β-actin (Ab-5) (Lab Vision, Fremont, CA, USA) for loading control.

### *In vitro* invasion assay

Polyvinylpyrrolidone-free polycarbonate filters, size 8 μm (Costar, Cambridge, MA, USA), were soaked in matrigel (1:5 in DMEM; BD Biosciences, Cockeysville, MD, USA) at 4°C for 36 h. The filters were then incubated at 37°C for 2 h, before being washed with DMEM and placed in microchemotaxis chambers. Cells were treated by 0.1% trypsin-EDTA digestion, resuspended in DMEM containing 10% FBS, and then washed with serum-free DMEM. Finally, cells (1 × 10^5^) were suspended in DMEM (200 μl) and placed in the upper compartment of the chemotaxis chambers. Culture medium (300 μl) containing 20% FBS was placed in the lower compartment of the chemotaxis chambers to serve as a source of chemoattractants. After incubation for 6 h (for the vector-transfected B16F10 cells) and 24 h (for the vector-transfected COLO 205 cells) at 37°C, cells on the upper surface of the filters were completely wiped away with a cotton swab. Cells on the lower surface of the filters were fixed in methanol, stained with hematoxylin and eosin, and then counted under a microscope. Cells invaded to the microchemotaxis chambers were also counted. The assays were repeated 3 times, and each assay consisted of 4 replicate filters for each cell line. For each replicate, the number of migrated tumor cells in 10 randomly selected fields was determined, and the counts were averaged.

### Animal metastasis models

We purchased C57BL/6 mice aged 6 to 7 weeks old (National Laboratory Animal Center, Taipei, Taiwan) and housed them in an animal holding room under standard conditions (22°C; 50% humidity; 12-h light/dark cycle). Each mouse was injected in the tail vein with viable cells (3 × 10^4^ cells in 100 μl PBS/mouse). The experiment included 11 and 14 mice injected with B16-EV and B16-CSE1L cells, respectively. Three weeks after injection, the mice were sacrificed and necropsied. The numbers of tumors in their lungs were counted using macrography and micrography.

For the animal liver metastasis model*,* NOD SCID mice (age range 6 to 7 weeks old; National Laboratory Animal Center, Taipei, Taiwan) were housed as described above. The experiment included eight mice injected with HT-29-EV cells and eight mice injected with HT-29-CSE1L cells. Mice were anesthetized using isofluorane, a small abdominal flank incision was created and the spleen was exteriorized. HT-29-EV or HT-29-CSE1L cells (3 × 10^6^ cells in 100 μl PBS/mouse) were injected into the spleen with a 27-gage needle, and the spleen was returned to the abdomen and the wound was closed. Three weeks after injection, mice were sacrificed. A complete autopsy with inspection of the liver, lung, kidneys, intestine, and spleen for macroscopically visible tumors was performed. Suspicious tissue lesions were examined histopathologically. Mouse care and experimental procedures were performed following the guideline of the Animal Care Committee of Academia Sinica, Taiwan.

### Patients

CRC samples were obtained from 128 consecutive human patients who had recently received a diagnosis at our hospital. The study protocol was approved by the Ethics Committee of the Taipei Medical University Hospital, Taipei, Taiwan. All participants had the study explained to them and gave informed consent by using institutional review board-approved guidelines before any participation in accordance with the guidelines approved by the institutional review board. The study group included 76 men and 52 women. The mean age of the patients was 64.3 years (range, 28 to 93 y). Tumor staging, grading, and categorization followed the 7^th^ edition of the Cancer Staging Manual of the American Joint Committee on Cancer [17] and the 7^th^ edition of the TNM Classification of Malignant Tumours [18]. In these systems, a grade 1 tumor is defined as well-differentiated, grade 2 is moderately differentiated, and grade 3 is a poorly differentiated tumor.

### Immunohistochemical tissue microarray

Three tissue cores from cancerous tissue and one tissue core from non-cancerous tissue in each paraffin block was longitudinally cut and arranged into new paraffin blocks to generate tissue microarrays. The manual method of the BiosynMatric Handmade Kit (Formosa Transcrip, Kaohsiung, Taiwan) was used. The tissue sections were stained with hematoxylin and eosin to confirm the presence of morphologically representative areas of the original cancers.

The paraffin-embedded CRC specimens and paired non-tumorous tissue sections (4 μm) were deparaffinized in xylene and rehydrated in graded alcohol. Antigen retrieval was performed by treatment with a boiling citrate buffer (10 mmol/L, pH 6.0) for 20 min. Endogenous peroxidase activity was blocked using 3% hydrogen peroxide in water, and nonspecific staining was blocked by incubation with 5% bovine serum albumin in PBS for 1 h at room temperature. The samples were incubated with an 100-fold dilution of anti-CSE1L antibody (clone 3D8, Abnova, Taipei, Taiwan) for 20 min at room temperature and were then washed 3 times with PBS; thereafter, the slides were incubated with a horseradish peroxidase/Fab polymer conjugate for another 30 min. The sites of peroxidase activity were visualized using diaminobenzidine (3, 3^′^-diaminobenzidine tetrahydrochloride) as the substrate and were counterstained using Mayer’s hematoxylin. In the negative control, the primary antibody was omitted and replaced by PBS. CRC tissues are known to be positive for CSE1L staining [12]; thus, no positive control was used in this study.

### Immunohistochemical scoring system

The results of CSE1L immunohistochemical staining were analyzed using a semiquantitative scoring system. The system combined the percentage of immunoreactive cells (quantity score) and an estimate of staining intensity (staining intensity score). Each tumor was scored according to the quantity and intensity of the nuclear or cytoplasmic staining, with the rating being confirmed by 2 expert pathologists. The proportion of cells stained was recorded as a percentage, and the intensity score was based on a scale of 0 to 3: 0, no staining; 1, weak staining; 2, moderate staining; and 3, strong staining. The total possible intensity score was 300, calculated as follows: (0 × percentage unstained) + (1 × percentage of weak staining) + (2 × percentage of moderate staining) + (3 × percentage of strong staining). We subdivided the CSE1L immunohistological staining results into low-CSE1L (CSE1L staining 0 and 1+) and high-CSE1L (CSE1L staining 2+ and 3+) subgroups.

### Statistical analysis

The between-groups differences for the clinicopathologic variables were assessed using the Fisher exact test. The prognostic ability of each variable was evaluated, including nuclear and cytoplasmic expressions of CSE1L, tumor grade, clinical stage, T status, and lymph node metastasis. The primary outcome was overall patient survival, which was defined as the time from surgery to death because of the disease, or to the date of the final follow-up. Disease-related death was confirmed by autopsy and imaging (computerized tomography and/or magnetic resonance imaging). Univariate analysis for overall survival used the Cox proportional hazards model and estimates of hazard ratios, and *P* values were adjusted for multiple significance. The distribution of overall survival was estimated using Kaplan-Meier analysis and log-rank tests. All analyses were performed using the Statistical Package for Social Sciences (SPSS) version 15.0 (SPSS, Inc, Chicago, IL, USA). A *P* value less than 0.05 (for a two-tailed test) was considered statistically significant.

## Results

### CSE1L overexpression increased invasion of colorectal cancer cells

**Figure 1:**
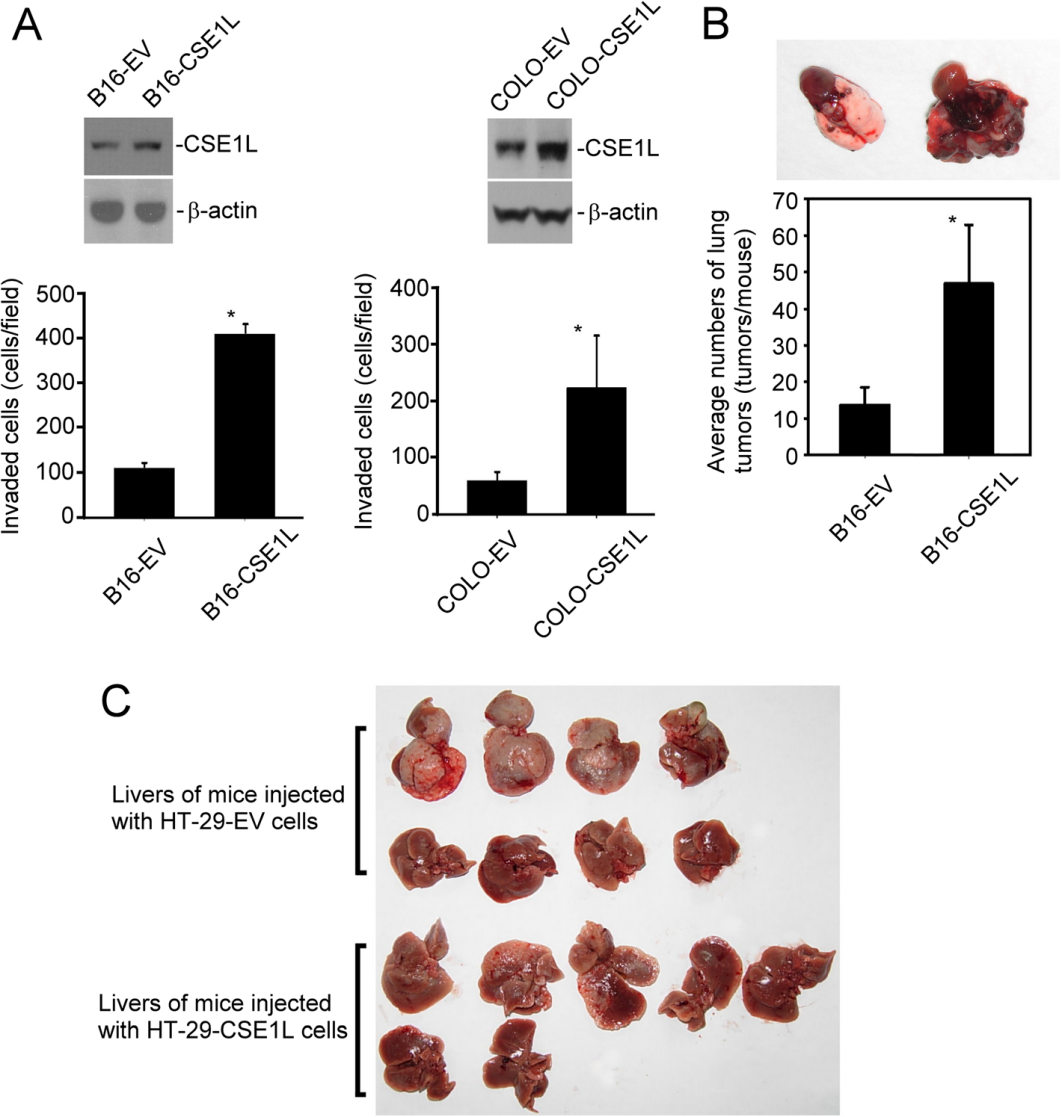
**Increased CSE1L expression enhanced the invasiveness and metastasis of cancer cells.** (**A**) The levels of CSE1L expression in B16-EV, B16-CSE1L, COLO-EV, and COLO-CSE1L cells were assayed by immunoblotting with anti-CSE1L antibody. The β-actin levels were assayed as a control. The invasive ability of the cells was analyzed by *in vitro* invasion assays using chemotaxis chambers, as described in “Materials and Methods”. (**B**) Animal models showed that CSE1L regulated the metastasis of B16F10 cancer cells. The upper figure is a representative photograph of pulmonary tumors in C57BL/6 mice injected with B16-EV and B16-CSE1L cells. (**C**) *In vivo* metastasis study showed that CSE1L regulated the hepatic metastasis of HT-29 colon cancer cells in nude mice. Eight mice were injected with HT-29-EV cells and eight mice were injected with HT-29-CSE1L cells. One mouse injected with HT-29-CSE1L cells died 3 days after injection was excluded from the study. Metastatic tumors in the livers of SCID mice were examined 21 days after injection.

We studied the effect of CSE1L expression on the invasion and metastasis of tumor cells. Previous studies have reported that malignant melanoma is a frequent source of metastasis to the gastrointestinal tract, including the colon [19]. The B16F10 melanoma cell line is widely used as a model for studying numerous aspects of cancer biology, including metastasis [20]. We used B16F10 melanoma cells and COLO 205 human colon cancer cells to study the invasion- and metastasis-stimulating activities of CSE1L in cancer. The B16F10 cells and COLO 205 cells were transfected with the control pcDNA empty vectors and the pcDNA-CSE1L vectors to yield B16-EV, B16-CSE1L, COLO-EV, and COLO-CSE1L cells, respectively (Figure 1A). Matrigel-based invasion assay showed that CSE1L overexpression was associated with increased *in vitro* invasiveness of B16F10 melanoma cells and COLO 205 colon cancer cells (Figure 1A). The cell count for invaded cells per field was 109.4 ± 12.6 (mean ± SD) for B16-EV cells and 406.8 ± 23.9 for B16-CSE1L cells. For COLO-EV and COLO-CSE1L cells, the count for invaded cells was 59.5 ± 15.2 and 222.5 ± 91.8, respectively. The animal-model study showed that CSE1L overexpression was associated with an increase in tumor pulmonary metastasis of the B16F10 melanoma cells. CSE1L overexpresion increased the tumor pulmonary metastasis of the B16F10 melanoma cells in C57BL/6 mice by 100% (*P*=0.01) (Figure 1B). The lung tumors were 13.7 ± 4.8 (mean ± SD, tumors per mouse) and 47 ± 15.8 for mice injected with B16-EV and B16-CSE1L cells, respectively.

*In vivo* metastasis experiments using human colon cancer cells overexpressing CSE1L were also performed to study the association between CSE1L expressing and the metastasis of colorectal cells to the liver, the main target organ for colorectal cancer. Injection of colorectal tumor cells directly into the spleen of nude mice has been reported to be a suitable animal model system to study colorectal tumor-cell metastasis [21]. The Colo 205 human colon cancer cells are reported to be unable to metastasize to the lung or liver of nude mice after intrasplenic injections in the colorectal tumor-cell metastasis experiments [21], thus we used the HT-29 human colon cancer cells to study the colorectal tumor *in vivo* metastasis experiments. SCID mice were intrasplenically injected with HT-29 cancer cells transfected with vectors expressing CSE1L (i.e. the HT-29-CSE1L cells) or HT-29 cancer cells transfected with the empty vectors (i.e. the HT-29-EV cells) [16]. Tumors in mice were examined and counted 21 days after injection. The proliferation rate of HT-29-CSE1L cells was slower than that of HT-29-EV cells due to the induction of cell polarity by CSE1L in HT-29 cells [16]. Nevertheless, our data showed that enhanced CSE1L expression increased the metastasis of HT-29 colon cancer cells to the livers of the mice, although obviously the tumor lesions were bigger in liver tumors induced by HT-29-EV cells than that induced by HT-29-CSE1L cells (Figure 1C). Liver metastasis were assayed in 71.4% (5/7) of mice injected with HT-29-CSE1L cancer cells, and were assayed in 50.0% (4/8) of mice injected with HT-29-EV cancer cells. No metastasis of the tumor cells to other organs was observed. The results indicate that CSE1L plays a role in regulating the metastasis of colorectal cancer cells.

### CSE1L expression was increased in colorectal carcinomas and cytoplasmic CSE1L was associated with T status and tumor stage

**Figure 2:**
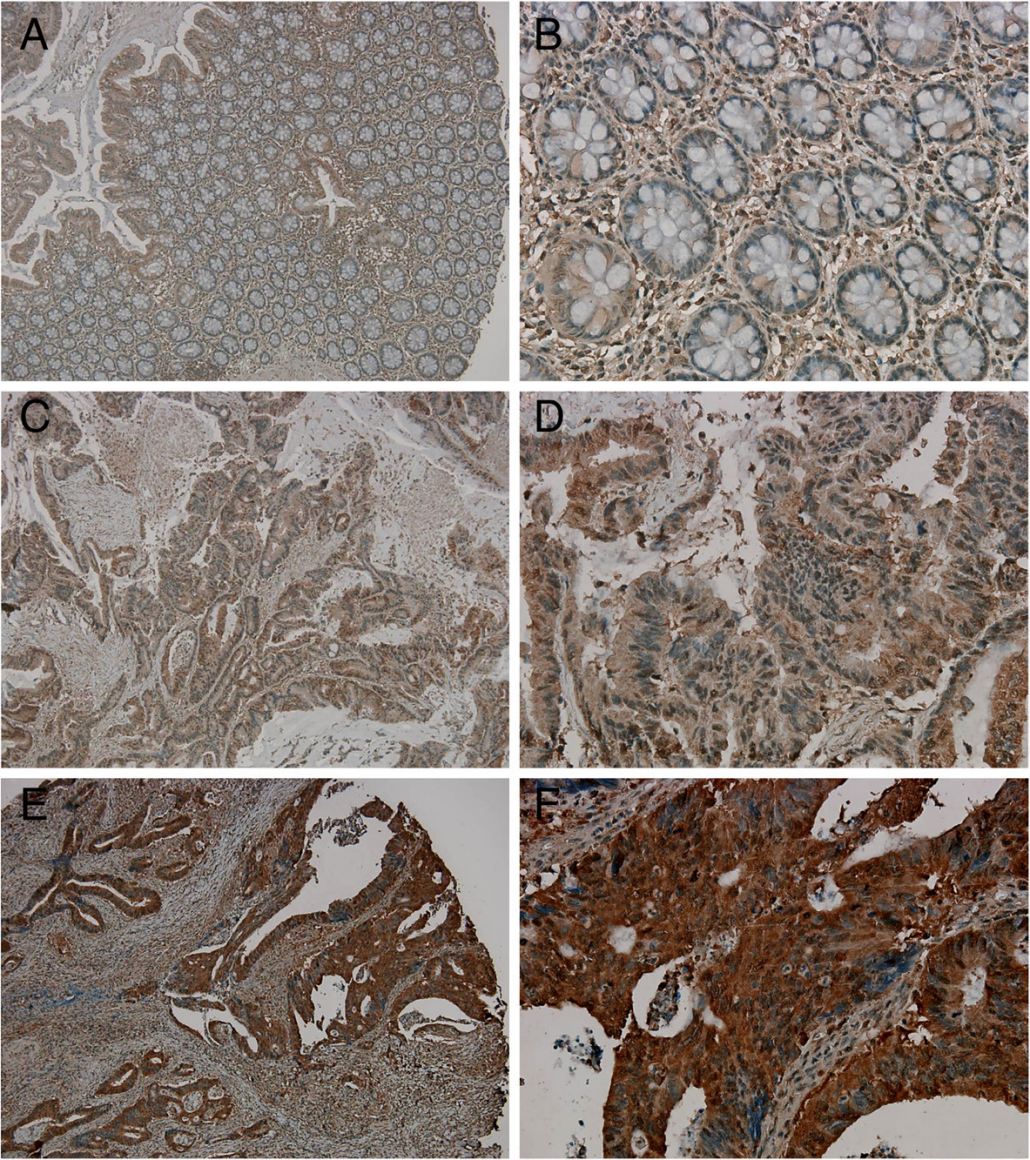
**CSE1L expression in non-tumorous colorectal glands and cytoplasmic CSE1L expression in colorectal carcinomas.** (**A**, **B**) Representative immunohistochemical images showed very low (±) CSE1L staining in nonneoplastic colorectal glands. (**C-F**) Results for CSE1L cytoplasmic staining in colorectal carcinoma displaying low (**C**, **D**) or high (**E**, **F**) staining. Original magnification: **A**, **C**, and **E**, 100×; **B**, **D**, and **F**, 400×.

**Figure 3:**
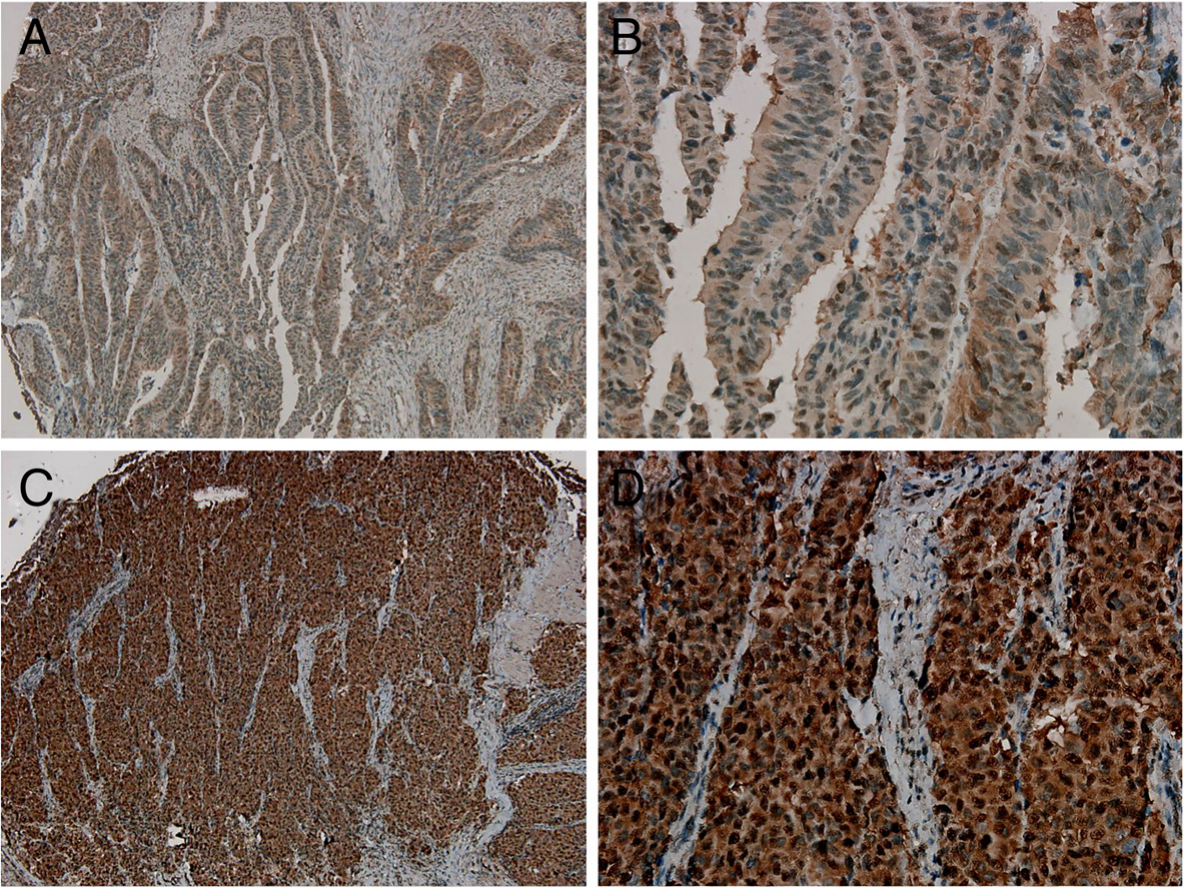
**Representative immunohistochemical images of nuclear CSE1L staining in colorectal carcinoma.** Colorectal tumor samples were classified as low-CSE1L nuclear staining (**A**, **B**) or high-CSE1L nuclear staining (**C**, **D**). Original magnification: **A** and **C**, 100×; **B** and **D**, 400×.

Colorectal glands are important functional organs in colorectal tissue and are also the origin of colorectal carcinomas [22]. We performed immunohistochemistry analysis with tissue microarray consists of 128 colorectal tumors and normal marginal tissues. The results showed that the non-neoplastic colorectal glands typically displayed very low (±) CSE1L staining (Figure 2). By contrast, most colorectal carcinomas (99.2%, 127/128) were significantly positive (1+ to 3+) for CSE1L staining, with both the cytoplasm and nuclei of tumor cells typically showing a positive stain (Figures 2 and 3). These results suggested that the use of immunohistochemical analysis of CSE1L expression in colorectal glands might be valuable in diagnosing CRC.

Expression of CSE1L in non-neoplastic colorectal glands was used as an internal control and provided a scoring baseline for CSE1L staining in colorectal tumors. The immunohistochemical data were analyzed using a semiquantitative scoring system for the quantity and intensity of nuclear or cytoplasmic CSE1L staining. Two subgroups were classified: high staining (2+ and 3+) and low staining (0 and 1+) (Figures 2 and 3). For cytoplasmic CSE1L staining, 63 cases displayed low staining (49.2%, 63/128), and 65 cases showed high staining (50.8%, 65/128) (Table 1). For nuclear CSE1L immunoreactivity, 70 cases displayed low staining (54.7%, 70/128) and 58 showed high staining (45.3%, 58/128) (Table 2). The associations between CSE1L immunohistochemical staining (cytoplasmic and nuclear) and the clinical parameters were analyzed, and the results are shown in Tables 1 and 2. The clinical parameters included tumor grade, T status, stage, lymph node metastasis, and overall survival.

**Table 1:** Tumor cytoplasmic CSE1L expression and clinical parameters in CRC

	**Cytoplasmic CSE1L**		**Total**	***P***
	**Low (1+)**	**High (2+ or 3+)**		
Sex				
female	26 (50)^#^	26 (50)	52	0.884
male	37 (48.7)	39 (51.3)	76	
Grade				
well	2 (50)	2 (50)	4	0.138
moderate	55 (47)	62 (53)	117	
poor	6 (85.7)	1 (14.3)	7	
T status				
T1+T2	16 (76.2)	5 (23.8)	21	0.007*
T3+T4	47 (43.9)	60 (56.1)	107	
Lymph node metastasis				
no	42 (56)	33 (44)	75	0.068
yes	21 (39.6)	32 (60.4)	53	
Distant metastasis				
no	56 (50.5)	55 (49.5)	111	0.476
yes	7 (41.2)	10 (58.8)	17	
Stage				
I	16 (80)	4 (20)	20	0.018*
II	22 (45.8)	26 (54.2)	48	
III	20 (45.5)	24 (54.5)	44	
IV	5 (31.3)	11 (68.8)	16	
Stage				
I/ II	38 (55.9)	30 (44.1)	68	0.108
III/IV	25 (41.7)	35 (58.3)	60	
Overall survival				
≤5 y	33 (44.6)	41 (55.4)	74	0.221
≥5 y	30 (55.6)	24 (44.4)	54	

Data are shown as number of cases (%).

* significant at <0.05.

** significant at <0.005.

**Table 2:** Tumor nuclear CSE1L expression and clinical parameters in CRC

	**Nuclear CSE1L**		**Total**	***P***
	**Low (1+)**	**High (2+ or 3+)**		
Sex				
female	33 (63.5) ^#^	19 (36.5)	52	0.099
male	37 (48.7)	39 (51.3)	76	
Grade				
well	3 (75)	1 (25)	4	0.699
moderate	63 (53.8)	54 (46.2)	117	
poor	4 (57.1)	3 (42.9)	7	
T status				
T1+T2	13 (61.9)	8 (38.1)	21	0.467
T3+T4	57 (53.3)	50 (46.7)	107	
Lymph node metastasis				
no	40 (53.3)	35 (46.7)	75	0.714
yes	30 (56.6)	23 (43.4)	53	
Distance metastasis				
no	62 (55.9)	49 (44.1)	111	0.497
yes	8 (47.1)	9 (52.9)	17	
Stage				
I	12 (60)	8 (40)	20	0.346
II	23 (47.9)	25 (52.1)	48	
III	28 (63.6)	16 (36.4)	44	
IV	7 (43.8)	9 (56.2)	16	
Stage				
I/ II	35 (51.5)	33 (48.5)	68	0.436
III/IV	35 (58.3)	25 (41.7)	60	
Overall survival				
≤5 y	39 (52.7)	35 (47.3)	74	0.579
≥5 y	31 (57.4)	23 (42.6)	54	

Data are shown as number of cases (%).

**Figure 4:**
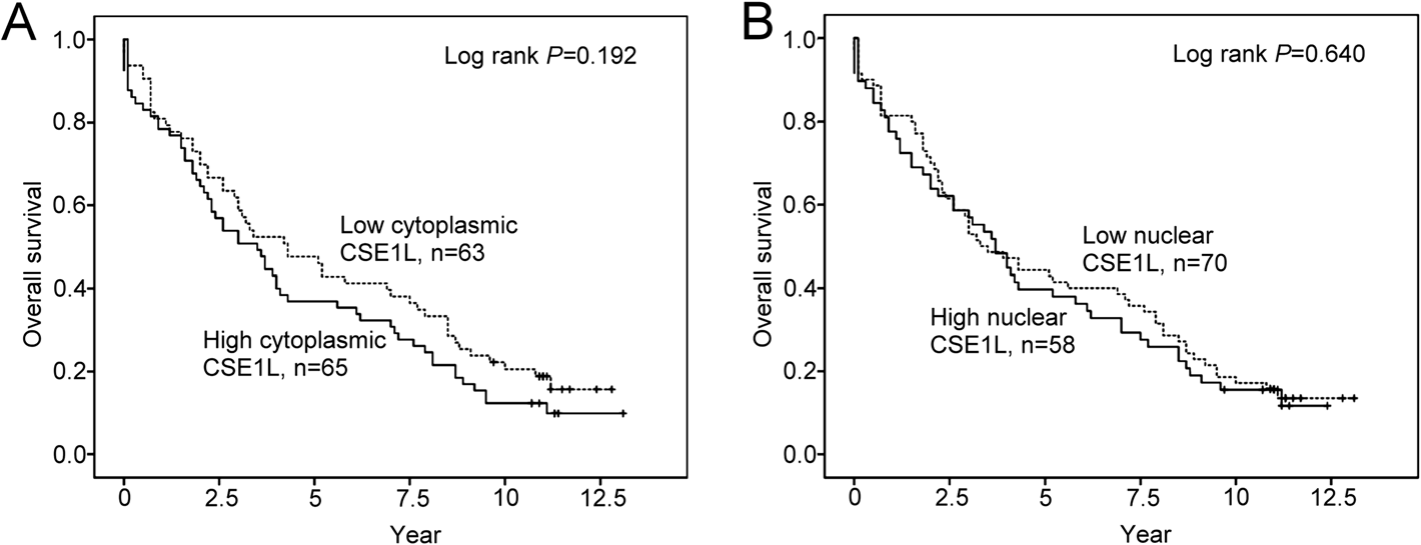
**Overall survival of colorectal cancer patients in relation to CSE1L expression.** Kaplan-Meier curves for the overall survival of patients with CRC, in relation to the degree of cytoplasmic (**A**) and nuclear (**B**) CSE1L immunohistochemical staining. Verticals marks indicate censored events.

Statistical analysis showed that high-CSE1L cytoplasmic staining in a CRC tumor was associated with advanced cancer stage (Stage II, III, or IV) and depth of tumor penetration (T status) (Table 1). No statistically significant relationship was noted between nuclear CSE1L staining and clinical manifestations, including tumor stage (Table 2). Analysis of the survival data showed that high cytoplasmic CSE1L staining in colorectal tumors was associated with a relatively poor overall patient survival rate, compared with cases displaying low staining (Figure 4). The median survival rate of cases displaying high cytoplasmic CSE1L expression was 42.0 mo, whereas in cases with low expression it was 51.6 mo. Similarly, the mean survival rate was 54.0 mo and 64.8 mo, respectively, for cases with high and low cytoplasmic CSE1L expression. For nuclear CSE1L expression, the median survival time for cases displaying high expression was 40.8 mo, compared with 44.4 mo for low expression. The mean survival rate was 56.4 mo and 61.2 mo, respectively, for high and low nuclear CSE1L expression cases. The Cox proportional hazards model (univariate analysis) showed that for patients with high cytoplasmic CSE1L expression, survival was significantly associated with tumor size, lymph node metastasis, and TNM stage (Table 3).

**Table 3:** Survival of CRC patients displaying high CSE1L cytoplasmic expression in tumor

	**Univariate Model**^**#**^		
**Variable**	**Hazard ratio**	**95% CI**	***P***
Cytoplasmic CSE1L			
Low	1	0.9 to 1.9	0.198
high	1.3		
T status			
T1+T2	1	1.4 to 3.5	<0.001***
T3+T4	2.2		
Lymph node metastasis			
no	1	1.3 to 2.4	<0.001***
yes	1.7		
Stage			
I/ II	1	1.3 to 2.5	<0.001***
III,IV	1.8		

^#^Cox proportional hazard model.

*** significant at < 0.001.

## Discussion

The diagnosis and prognosis of CRC may be challenging at times. Although patients with stage I tumors usually have a good prognosis, some suffer local recurrence after curative resection [23]. Approximately 10% to 30% of patients with stage II disease have experienced recurrence of this cancer [24]. Early detection and treatment of colonic adenomas and early-stage CRC can significantly reduce the incidence and mortality rate of CRC [25]. Several studies have shown that the use of biomarkers is a valuable aid to the diagnosis and prognosis of this disease, and enhances decision-making about treatment [26-28]. Our data showed that CSE1L was positively stained (1+ to 3+) in most of the colorectal carcinomas (99.2%), whereas the non-neoplastic colorectal glands showed relatively low (±) CSE1L staining. Therefore, CSE1L may be a useful immunohistochemical marker for the detection and diagnosis of CRC.

This study showed that CSE1L overexpression was associated with increased invasion and metastasis in B16F10 melanoma cells, COLO 205 human CRC cells, and HT-29 human CRC cells (Figure 1). We had reported previously that CSE1L overexpression in HT-29 human CRC cells stimulated the polarity of these cells and inhibited their migration [16,29]. These results contradict the findings of the current study, in which CSE1L expression was associated with increased invasion and metastasis of tumor cells. However, the HT-29 cell line appears to be unusual. In our previous studies on CSE1L expression in other cancer cells, CSE1L overexpression did not stimulate the polarity of various cell lines, including B16F10 melanoma cells and MCF-7 breast cancer cells [15,30]. Furthermore, CSE1L overexpression did not inhibit the tumorigenicity of these cells [15,30]. Overexpression of CSE1L in B16F10 melanoma cells and MCF-7 breast cancer cells stimulated invadopodia extension and matrix metalloproteinase-2 (MMP-2) secretion and increased the migration and invasiveness of the cancer cells [15,30]. Similarly, CSE1L overexpression in COLO 205 colon cancer cell line was associated with an increase in the invasive ability of the cells (Figure 1). The HT-29 human CRC cell line is a special cell line that readily develops polarity in an *in vitro* cell culture [31]. Thus, the association between CSE1L overexpression and the development of polarity and tumorigenicity inhibition observed in HT-29 cancer cells appears to be specific to that cell line. We cannot exclude the possibility that CSE1L may play a role in polarity formation during embryo development [32]. In the animal liver metastasis model, although the tumor lesions induced by HT-29-CSE1L cells were obviously smaller than that tumor lesions induced by HT-29-EV cells, reflecting the lower proliferation rate of HT-29-CSE1L cells due to the induction of cell polarity by CSE1L in HT-29 cells [16]. Nevertheless, the data showed that enhanced CSE1L expression increased the *in vivo* metastasis of HT-29 colon cancer cells to the livers of the SCID mice (Figure 1C). The results indicate that CSE1L plays a role in regulating the metastasis of colorectal cancer cells. Moreover, CSE1L is known to stimulate the extension of invadopodia and the secretion of matrix metalloproteinases of several cancer cell lines [15,33]. Thus, stimulation of tumor invasion and metastasis should be the correct role of CSE1L in tumor development.

Our results also showed that in cases of CRC displaying high CSE1L cytoplasmic staining, the degree of staining was associated with the T status and disease stage. Tumor stage is significantly associated with clinical outcomes in CRC patients, and the 5-y survival rate decreases as the stage advances. Most deaths from CRC are directly related to an advanced stage of the disease [34,35]. Our results showed that although high cytoplasmic CSE1L staining in colorectal tumors was associated with a lower overall survival rate compared with cases displaying low cytoplasmic CSE1L expression, the difference was not statistically significant (Figure 4). Alnabulsi *et al*. reported that CSE1L expression was related to the occurrence of lymph node metastasis and the overall survival rate of patients with CRC [36]. Their survival analysis showed that early-stage colorectal cancer patients whose tumors showed high CSE1L cytoplasmic expression experienced poor survival outcomes [36]. The inconsistency in our results might be attributable to the problems in staging CRC accurately using the current evaluation methods, especially in the early stage where occult metastasis may occur. Lymphatic invasion in malignant polyps of the colon and rectum was reported to be associated with an increased risk of regional lymph node metastases [37]. Similarly, venous invasion in colorectal carcinoma was recognized as a risk factor for liver metastases [38]. We have studied the relationship between CSE1L expression and tumor cell lymphatic vessel invasion and blood vessel invasion, using 60 whole-mount sections of CRC specimens with antibodies against the D2-40 lymphatic endothelial marker and the CD31 blood vessel endothelial marker. The results showed that lymphatic or blood vessel invasion was present in sixteen cases (26.6%), but there was no statistic correlation between CSE1L expression and tumor cell lymphatic vessel invasion and blood vessel invasion (data not shown). However, at present there are still many problems in the assay of lymphatic and blood vessel invasion in CRC, such as the variability in recognition, diagnosis, and reporting of lymphovascular invasion, as well as in processing of specimens [39]. For example, the result from a single focus on a single slide is insufficient to confirm the absence of lymphovascular invasion in a tumor. Therefore, the relationship between CSE1L expression and colorectal cancer lymphatic and blood vessel invasion still need to be investigated.

As mentioned, cytoplasmic CSE1L is associated with the development of microtubules, and this association may stimulate invadopodia extension and may enhance the migration of tumor cells [15]. Previous studies on cancer cells showed that CSE1L was colocalized with MMP-2 in cytoplasmic areas near the cell membranes and invadopodia, and that CSE1L overexpression enhanced MMP-2 secretion and the invasive ability of cancer cells [15,40,41]. Furthermore, a reduction in CSE1L expression inhibited the metastasis of tumor cells in animal models [15,40,41]. Microvesicles are rich in metastasis-related proteases and play a role in the interaction between tumor cells and the tumor microenvironment during metastasis [42]. A recent report on B16F10 melanoma cells showed that CSE1L overexpression triggered microvesicle generation, whereas CSE1L knockdown diminished Ras-induced microvesicle generation, MMP-2/MMP-9 secretion, and metastasis [33]. Recent studies have proposed that secretory CSE1L might be a potential prognostic marker of cancer [33,40,41,43]. This study further showed that cytoplasmic CSE1L was involved in the invasiveness of CRC cells. Thus, analysis of the cytoplasmic distribution of CSE1L might provide a valuable tool for diagnosing CRC and determining the prognosis.

## Conclusion

This study showed that for patients with CRC, cytoplasmic CSE1L expression in neoplastic colorectal glands correlated with depth of tumor penetration and cancer stage. Analysis of the nuclear and cytoplasmic distribution of CSE1L in colorectal tumors might provide valuable clinical-pathological information to aid physicians in making decisions about treatment. Ultimately, this could reduce the mortality from the disease.

## Abbreviations

DMEM: Dulbecco’s modified Eagle’s medium; ECL: Enhanced chemiluminescence; EDTA: Ethylenediaminetetraacetic acid; FBS: Fetal bovine serum; MMP-2: Matrix metalloproteinase-2; PBS: Phosphate buffered saline; RIPA: Radio-immunoprecipitation assay; SDS: Sodium dodecyl sulfate.

## Authors’ contributions

CHC provided the surgical specimens, and SKH, YCM, YKT, and STC performed the immunohistochemical analysis and evaluated the results. JMC, LLT, SSC, and LWR conducted the additional experiments. LCF, CYC, and SHY performed the animal studies. LSH and LCH performed the statistical analysis. JMC, TCJ, and CCC designed the study and wrote the manuscript, and all authors read and approved the final manuscript.
