# Correction: Correlations between cytoplasmic CSE1L in neoplastic colorectal glands and depth of tumor penetration and cancer stage

**DOI:** 10.1186/s12967-023-04148-w

**Published:** 2023-05-09

**Authors:** Cheng-Jeng Tai, Tzu-Cheng Su, Ming-Chung Jiang, Hung-Chang Chen, Shing-Chuan Shen, Woan-Ruoh Lee, Ching-Fong Liao, Ying-Chun Chen, Shu-Hui Lin, Li-Tzu Li, Ko-Hung Shen, Chung-Min Yeh, Kun-Tu Yeh, Ching-Hsiao Lee, Hsin-Yi Shih, Chun-Chao Chang

**Affiliations:** 1 Department of Internal Medicine, School of Medicine, College of Medicine, Taipei Medical University Hospital, No. 250, Wu-Hsing St., Taipei, 11031 Taiwan; 2 Division of Hematology and Oncology, Department of Internal Medicine, Taipei Medical University Hospital, No. 250, Wu-Hsing St., Taipei, 11031 Taiwan; 3 Department of Pathology, Changhua Christian Hospital, 135 Nan-Hsiao St., Changhua, 50006 Taiwan; 4 Division of Colorectal Surgery, Changhua Christian Hospital, No. 135 Nan-Hsiao St., Changhua, 50006 Taiwan; 5 Nursing and Management, Jen-Teh Junior College of Medicine, No. 79-9, Shalunhu, Houlong Township, Miaoli, 35664 Taiwan; 6 Graduate Institute of Medical Sciences, College of Medicine, Taipei Medical University, No. 252, Wu-Hsing St., Taipei, 11031 Taiwan; 7 Institute of Cellular and Organismic Biology, Academia Sinica, No. 128 Academia Road, Section 2, Nankang, Taipei, 11529 Taiwan; 8 School of Medicine, Chung Shan Medical University, No. 110, Sec.1, Jianguo N.Rd., Taichung, 40201 Taiwan; 9 Institute of Clinical Medicine, Kaohsiung Medical University, No. 100, Shih-Chuan 1st Road, Kaohsiung, 80708 Taiwan; 10 Division of Gastroenterology and Hepatology, Department of Internal Medicine, Taipei Medical University Hospital, No. 250, Wu-Hsing St., Taipei, 11031 Taiwan

**Correction: Journal of Translational Medicine (2013) 11:29** 10.1186/1479-5876-11-29

Since the publication of our article [1], it has come to our attention that the legend to Fig. 1(A) left panel did not acknowledge that this figure had been adapted from a published source. The legend to Fig. 1(A) should read as follows:

(A) The levels of CSE1L expression in B16-EV, B16-CSE1L, COLO-EV, and COLO-CSE1L cells were assayed by immunoblotting with anti-CSE1L antibody. The β-actin levels were assayed as a control. The invasive ability of the cells was analyzed by in vitro invasion assays using chemotaxis chambers, as described in “Materials and Methods”. The left panel is adapted with permission from Fig. 5C in Liao CF, Luo SF, Li LT, Lin CY, Chen YC, Jiang MC. CSE1L/CAS, the cellular apoptosis susceptibility protein, enhances invasion and metastasis but not proliferation of cancer cells. J Exp Clin Cancer Res. 2008; 27:15. https://jeccr.biomedcentral.com/articles/10.1186/1756-9966-27-15. Copyright © 2008, Liao et al.; licensee BioMed Central Ltd.
